# Supplementary material for: The interaction between rumination and job embeddedness among trainee nurses during internship: A multicenter longitudinal study
Source: PLoS One. 2026 May 26;21(5):e0349402. doi: 10.1371/journal.pone.0349402 (PMC13210194; doi:10.1371/journal.pone.0349402)
Supplement: Attachment — Submitted filename: PONE-D-25-60904 (R).pdf [file pone.0349402.s002.pdf]

PLOS ONE

The interaction between rumination and job embeddedness among trainee nurses duringinternship: a multicenter longitudinal study

--Manuscript Draft--

**Manuscript Number:**	PONE-D-25-60904
**Article Type:**	Research Article
**Full Title:**	The interaction between rumination and job embeddedness among trainee nurses duringinternship: a multicenter longitudinal study
**Short Title:**	a multicenter longitudinal study
**Corresponding Author:**	weiyi wang<br>Nanjing Drum Tower Hospital: Nanjing University Medical School Affiliated Nanjing Drum Tower Hospital<br>Nanjing, CHINA
**Keywords:**	Trainee nurses; Internship period; Rumination; Job embeddedness; Relevance; Cross-lag model
**Abstract:**	Objective<br><br>To explore the developmental trajectory of rumination and job embeddedness among college trainee nurses and examine the predictive relationship between them, with the aim of providing a theoretical basis for improving trainee nurses' job embeddedness.<br><br>Methods<br><br>Selecting 12 Chinese colleges and universities offering degrees at or above the college level, 405 cases of practical trainee nurses were chosen as subjects for investigation. The study involved three time points: T1 (early stage of internship, 1–2 months), T2 (middle stage, 4–5 months), and T3 (end stage, August to October). Rumination and job embeddedness were tracked over time. Data were analyzed using a cross-lagged model (CLM).<br><br>Results<br><br>The recycling of questionnaire 405 proved to be effective, with a recovery rate of 93.53%. In analyzing the overall trend from T1 to T3, rumination significantly decreased from a baseline of (50.21±5.14) to (43.27±5.23) (F=48.050, P<0.001). Concurrently, the score of job embeddedness increased significantly from (20.05±3.10) to (25.36±3.88) (F=25.714, P<0.001). The level of rumination at a previous time point negatively predicted job embeddedness at the subsequent point (β=-0.403, P < 0.01; β=-0.411, P < 0.001), and job embeddedness at a previous time point negatively predicted rumination at the next point on average (β=-0.397, P < 0.001; β=-0.385, P < 0.001). At the initial level, rumination can predict job embeddedness negatively (β=0.433, P=0.002), the initial level of rumination can predict the development speed of job embeddedness negatively (β=0.395, P=0.005), and the development speed of rumination can positively predict the development speed of job embeddedness (β=0.450, P < 0.001).<br><br>Conclusion<br><br>practice trainee nurses rumination is on the decline, job embeddedness is on the rise. Educators and clinical instructors should assess and monitor rumination levels in trainee nurses and strategically leverage the dynamic relationship between rumination and job embeddedness to enhance the latter during clinical training.
**Order of Authors:**	Weiyi Wang
	Huizhen Ma
	Lei Huang

Powered by Editorial Manager® and ProduXion Manager® from Aries Systems Corporation

	Chen Ying
**Additional Information:**	
**Question**	**Response**
**Financial Disclosure**<br><br>Enter a financial disclosure statement that describes the sources of funding for the work included in this submission. Review the submission guidelines for detailed requirements. View published research articles from *PLOS ONE* for specific examples.<br><br>This statement is required for submission and **will appear in the published article** if the submission is accepted. Please make sure it is accurate.<br><br>**Funded studies**<br>Enter a statement with the following details:<br>• Initials of the authors who received each award<br>• Grant numbers awarded to each author<br>• The full name of each funder<br>• URL of each funder website<br>• Did the sponsors or funders play any role in the study design, data collection and analysis, decision to publish, or preparation of the manuscript?<br><br>Did you receive funding for this work?	Yes
Please add funding details.<br>as follow-up to "**Financial Disclosure**<br><br>Enter a financial disclosure statement that describes the sources of funding for the work included in this submission. Review the submission guidelines for detailed requirements. View published research articles from *PLOS ONE* for specific examples.<br><br>This statement is required for submission and **will appear in the published article** if the submission is accepted. Please make sure it is accurate.	The study was supported by Project of China Hospital Reform and Development Research Institute of Nanjing University ,Nanjing Drum Tower Hospital and Aid project of Jiangsu Ningai Medical Development & Medical Aid Foundation(NDYGN2024064).

**Funded studies**<br>Enter a statement with the following details:<br>• Initials of the authors who received each award<br>• Grant numbers awarded to each author<br>• The full name of each funder<br>• URL of each funder website<br>• Did the sponsors or funders play any role in the study design, data collection and analysis, decision to publish, or preparation of the manuscript?<br><br>Did you receive funding for this work?"	
Please select the country of your main research funder (please select carefully as in some cases this is used in fee calculation).<br>as follow-up to "**Financial Disclosure**<br><br>Enter a financial disclosure statement that describes the sources of funding for the work included in this submission. Review the submission guidelines for detailed requirements. View published research articles from *PLOS ONE* for specific examples.<br><br>This statement is required for submission and **will appear in the published article** if the submission is accepted. Please make sure it is accurate.<br><br>**Funded studies**<br>Enter a statement with the following details:<br>• Initials of the authors who received each award<br>• Grant numbers awarded to each author<br>• The full name of each funder<br>• URL of each funder website<br>• Did the sponsors or funders play any role in the study design, data collection and analysis, decision to publish, or preparation of the manuscript?<br><br>Did you receive funding for this work?"	CHINA - CN
**Competing Interests**	The authors have no conflicts of interest to disclose.

Use the instructions below to enter a competing interest statement for this submission. On behalf of all authors, disclose any competing interests that could be perceived to bias this work—acknowledging all financial support and any other relevant financial or non-financial competing interests.<br><br>This statement is required for submission and **will appear in the published article** if the submission is accepted. Please make sure it is accurate and that any funding sources listed in your Funding Information later in the submission form are also declared in your Financial Disclosure statement.<br><br>View published research articles from *PLOS ONE* for specific examples.<br><br>**NO authors have competing interests**<br><br>Enter: *The authors have declared that no competing interests exist.*<br><br>**Authors with competing interests**<br><br>Enter competing interest details beginning with this statement:<br><br>*I have read the journal's policy and the authors of this manuscript have the following competing interests: [insert competing interests here]*<br><br>* typeset	
**Ethics Statement**<br><br>Enter an ethics statement for this submission. This statement is required if the study involved:<br><br>• Human participants<br>• Human specimens or tissue<br>• Vertebrate animals or cephalopods<br>• Vertebrate embryos or tissues	This study was finally approved by the Ethics Committee of Nanjing Drum Tower Hospital(ID: 2023-0124-02)

• Field research<br><br>Write "N/A" if the submission does not require an ethics statement.<br><br>General guidance is provided below. Consult the submission guidelines for detailed instructions. **Make sure that all information entered here is included in the Methods section of the manuscript.**	

**Format for specific study types**<br><br>**Human Subject Research (involving human participants and/or tissue)**<br>• Give the name of the institutional review board or ethics committee that approved the study<br>• Include the approval number and/or a statement indicating approval of this research<br>• Indicate the form of consent obtained (written/oral) or the reason that consent was not obtained (e.g. the data were analyzed anonymously)<br><br>**Animal Research (involving vertebrate animals, embryos or tissues)**<br>• Provide the name of the Institutional Animal Care and Use Committee (IACUC) or other relevant ethics board that reviewed the study protocol, and indicate whether they approved this research or granted a formal waiver of ethical approval<br>• Include an approval number if one was obtained<br>• If the study involved *non-human primates*, add *additional details* about animal welfare and steps taken to ameliorate suffering<br>• If anesthesia, euthanasia, or any kind of animal sacrifice is part of the study, include briefly which substances and/or methods were applied<br><br>**Field Research**<br><br>Include the following details if this study involves the collection of plant, animal, or other materials from a natural setting:<br>• Field permit number<br>• Name of the institution or relevant body that granted permission	
**Data Availability**<br><br>Authors are required to make all data underlying the findings described fully available, without restriction, and from the time of publication. PLOS allows rare exceptions to address legal and ethical concerns. See the PLOS Data Policy and FAQ for detailed information.	Yes - all data are fully available without restriction

Powered by Editorial Manager® and ProduXion Manager® from Aries Systems Corporation

A Data Availability Statement describing where the data can be found is required at submission. Your answers to this question constitute the Data Availability Statement and **will be published in the article**, if accepted.<br><br>**Important:** Stating 'data available on request from the author' is not sufficient. If your data are only available upon request, select 'No' for the first question and explain your exceptional situation in the text box.<br><br>Do the authors confirm that all data underlying the findings described in their manuscript are fully available without restriction?	
**Describe where the data may be found in full sentences. If you are copying our sample text, replace any instances of XXX with the appropriate details.**<br><br>• If the data are **held or will be held in a public repository**, include URLs, accession numbers or DOIs. If this information will only be available after acceptance, indicate this by ticking the box below. For example: *All XXX files are available from the XXX database (accession number(s) XXX, XXX.).*<br>• If the data are all contained **within the manuscript and/or Supporting Information files**, enter the following: *All relevant data are within the manuscript and its Supporting Information files.*<br>• If neither of these applies but you are able to provide **details of access elsewhere**, with or without limitations, please do so. For example:<br><br>*Data cannot be shared publicly because of [XXX]. Data are available from the XXX Institutional Data Access / Ethics Committee (contact via XXX) for researchers who meet the criteria for access to confidential data.*<br><br>*The data underlying the results presented in the study are available from (include the name of the third party*	All relevant data are within the manuscript and its Supporting Information files.

and contact information or URL).<br>• This text is appropriate if the data are owned by a third party and authors do not have permission to share the data.<br><br>* typeset	
Additional data availability information:	Tick here if the URLs/accession numbers/DOIs will be available only after acceptance of the manuscript for publication so that we can ensure their inclusion before publication.; Tick here if your circumstances are not covered by the questions above and you need the journal's help to make your data available.

Powered by Editorial Manager® and ProduXion Manager® from Aries Systems Corporation

Manuscript Click here to access/download;Manuscript;5.20SCI article.docx

The interaction between rumination and job embeddedness among trainee nurses during internship: a multicenter longitudinal study

Weiyi Wang[1], Huizhen Ma[1], Lei Huang[1], Chen Ying*[2]

WW and HM contributed equally to this work

Weiyi Wang (e-mail: anotherweiyi@163.com)

[1]Department of Geriatric Medicine, Nanjing Drum Tower Hospital: The Affiliated Hospital of Nanjing University Medical School, Nanjing, China

[2]Phase I Clinical Trials Unit, Nanjing Drum Tower Hospital: The Affiliated Hospital of Nanjing University Medical School, Nanjing, China

Correspondence: Chen Ying, Phase I Clinical Trials Unit, Nanjing Drum Tower Hospital: The Affiliated Hospital of Nanjing University Medical School, Nanjing, China (e-mail: 13814021690@163.com)

Abstract

Objective To explore the developmental trajectory of rumination and job embeddedness among college trainee nurses and examine the predictive relationship between them, with the aim of providing a theoretical basis for improving trainee nurses' job embeddedness.

Methods Selecting 12 Chinese colleges and universities offering degrees at or above the college level, 405 cases of practical trainee nurses were chosen as subjects for investigation. The study involved three time points: T1 (early stage of internship, 1–2 months), T2 (middle stage, 4–5 months), and T3 (end stage, August to October). Rumination and job embeddedness were tracked over time. Data were analyzed using a cross-lagged model (CLM).

Results The recycling of questionnaire 405 proved to be effective, with a recovery rate of 93.53%.

In analyzing the overall trend from T1 to T3, rumination significantly decreased from a baseline of (50.21±5.14) to (43.27±5.23) ($F=48.050$, $P<0.001$). Concurrently, the score of job embeddedness increased significantly from (20.05±3.10) to (25.36±3.88) ($F=25.714$, $P<0.001$). The level of rumination at a previous time point negatively predicted job embeddedness at the subsequent point ($\beta=-0.403$, $P < 0.01$; $\beta=-0.411$, $P < 0.001$), and job embeddedness at a previous time point negatively predicted rumination at the next point on average ($\beta=-0.397$, $P < 0.001$; $\beta=-0.385$, $P < 0.001$). At the initial level, rumination can predict job embeddedness negatively ($\beta=0.433$, $P=0.002$), the initial level of rumination can predict the development speed of job embeddedness negatively ($\beta=0.395$, $P=0.005$), and the development speed of rumination can positively predict the development speed of job embeddedness ($\beta=0.450$, $P < 0.001$).

Conclusion practice trainee nurses rumination is on the decline, job embeddedness is on the rise. Educators and clinical instructors should assess and monitor rumination levels in trainee nurses and strategically leverage the dynamic relationship between rumination and job embeddedness to enhance the latter during clinical training.

Key words Trainee nurses; Internship period; Rumination; Job embeddedness; Relevance; Cross-lag model

Introduction

With the rapid development of medical and healthcare services, the importance of nursing work has become increasingly prominent. Nursing has evolved from providing basic disease care to offering comprehensive services that encompass emotional support, health promotion, and environmental maintenance. Consequently, nurses' professional literacy and attitudes have a profound impact on Participants' rehabilitation experiences and quality of life [1, 2].

Rumination is a psychological concept referring to a cognitive pattern in which individuals repeatedly and passively dwell on negative emotions and the events that trigger them. It is characterized by persistent recall of personal mistakes and failures, accompanied by self-criticism, helplessness, and a perceived loss of control [3]. During clinical practice, trainee nurses are exposed to high-intensity stressors such as life-and-death situations and medical disputes for the first time [4]. The transition from student to clinical nurse demands rapid role adaptation and accountability for patient care. However, limited clinical proficiency often results in hesitation during decision-making, making trainee nurses particularly susceptible to rumination during the practice stage [5].

Research has shown that the correlation between persistent rumination and anxiety or depression is 2.3 times higher in trainee nurses than in the general population, as negative thoughts consume attentional resources [6]. One study found that trainee nurses with a high tendency toward rumination demonstrated a 19% decrease in clinical performance accuracy and a 41% lower professional identity score compared to the control group [7]. More critically, such cognitive patterns may lead to learned helplessness, causing some trainee nurses to develop avoidance behaviors in the later stages of their internship. This not only reduces their job embeddedness but also increases turnover intention, thereby hindering the development of a robust nursing talent echelon [8, 9].

Understanding the developmental trajectory of rumination and job embeddedness during clinical practice, and implementing timely psychological interventions for those with high rumination levels, can help mitigate negative emotions and enhance job embeddedness [10]. A dynamic examination of the patterns and interactions between these variables enables identification of key

intervention points, which can reduce rumination and foster stronger job embeddedness. This is crucial for enhancing the professional identity of trainee nurses and ensuring the delivery of high-quality nursing care.

Therefore, this study examines the trajectories and predictive relationships between rumination and job embeddedness among trainee nurses employing a longitudinal design using cross-lagged model (CLM) and latent growth models (LGM). The study aims to provide a theoretical foundation for university and clinical instructors seeking to improve trainee nurses' job embeddedness.

Research methods

Research subjects

This study employed a multicenter longitudinal survey design. From April 1, 2023, to May 30, 2024, a total of 405 trainee nurses from 12 colleges and universities in China were recruited as participants. Prior to data collection, ethical approval was obtained from the Ethics Committee of Nanjing Drum Tower Hospital. Questionnaires were distributed only after receiving approval. The inclusion criteria were as follows: (1) Trainee nurses who had completed their on-campus coursework and had entered clinical practice; (2) All participants signed an electronic written informed consent form and voluntarily participated in the questionnaire survey of this study. The exclusion criteria were as follows: (1) Trainee nurses who had been away from clinical practice for more than one-month (e.g., due to sick leave); (2) Questionnaires missing data from more than one time point. Based on the sample size requirements of the LGM, at least 200 cases were needed. Considering potential attrition, the sample size was adjusted using the formula n = 200/ (1 - 20%),

resulting in a minimum of 250 participants. Ultimately, 405 trainee nurses were included.

Survey tools

General information questionnaire

This self-designed questionnaire collected participants' demographic information, including their highest education level and the classification level of the hospital where they conducted their clinical practice.

Ruminative responses scale (RRS)

The Chinese version of the RRS, revised by Han Xiu et al.[11] consists of 22 items divided into three dimensions: brooding (5 items), symptom rumination (12 items), and reflection (5 items). Responses are rated on a 4-point Likert scale ranging from 1 ("never") to 4 ("always"), with a total score range of 22–88. Higher scores indicate greater rumination tendency. In this study, the Cronbach's α coefficients for the overall scale and the three dimensions were 0.880, 0.845, 0.880, and 0.832, respectively.

Global job embeddedness items (GJEI)

Originally developed by Crossley et al. and later translated and adapted by Meihua et al., the GJEI consists of 7 items measuring job embeddedness on a single dimension. It uses a 5-point Likert scale ranging from 1 ("strongly disagree") to 5 ("strongly agree"). Items 4 and 6 are reverse scored. Higher scores reflect greater job embeddedness. In this study, the Cronbach's α coefficients at different time points were 0.885, 0.812, and 0.844.

Data collection methods

Data were collected using a self-developed electronic questionnaire distributed via the WenJuanxing platform (Survey ID: 23045560). The questionnaire included demographic items and the two core scales (RRS and GJEI). Participation was voluntary, and the study strictly adhered to the principle of informed consent. A diachronic tracking design was adopted, with three data collection points: T1: Early stage of internship (1-2 months); T2: Middle stage (5-6 months); T3: End stage (9-10 months). The questionnaire platform was configured with a mandatory response mechanism, ensuring that all questions were completed before submission.

Statistical methods

Data analysis was conducted using SPSS 26.0 and Mplus 8.0. Categorical variables were presented as frequencies and percentages, while continuous variables were expressed as mean ± standard deviation. Pearson correlation analysis was used to examine associations between variables. To explore the linear developmental trajectory of rumination and job embeddedness, an unconditional linear LGM was used. The intercept represented the initial level, and the slope reflected the rate of change. A parallel growth model was employed to analyze the dynamic relationship between rumination and job embeddedness. Additionally, a CLM was used to assess the bidirectional predictive relationship between the two variables. Model fit was evaluated using the following indices: Chi-square/degrees of freedom ratio (χ^2/df); Comparative Fit Index (CFI); Tucker–Lewis Index (TLI); Root Mean Square Error of Approximation (RMSEA); Standardized Root Mean Square Residual (SRMR). Good model fit was indicated by χ^2df < 5.000, CFI > 0.900, TLI > 0.900,

RMSEA < 0.080, and SRMR < 0.100. A p-value of < 0.05 was considered statistically significant.

Results

General demographic data

A total of 382 valid questionnaires were collected, yielding an effective response rate of 94.32%. The respondents were college trainee nurses aged 19 to 27 years, including 43 males (11.26%) and 339 females (88.74%). Detailed demographic information is presented in Table 1.

Table 1 General information of the respondents (*n*=382)

Items	Categories	*n*	%	Items	Categories	*n*	%
Age (years)	< 20	45	11.78	Level of practice hospital	Grade II and below	25	6.54
	20 ~ 25	295	77.23		3rd Grade B	56	14.66
	> 25	42	10.99		3rd Grade A	301	78.80
Gender	Male	43	11.26	Whether he is an only child	Yes	96	25.13
	Female	339	88.74		No	286	74.87
Highest degree	College or below	95	24.87	Place of residence	City	100	26.18
	Undergraduate	245	64.14		Township	153	40.05
	Master's degree or	42	10.99		Rural	129	33.77

above

Common method deviation test

There were 12, 11, and 10 factors with eigenvalues greater than 1 in the T1, T2, and T3 tests, respectively. The explained variance for the three measurements was 15.18%, 18.55%, and 22.37%, all of which were below the critical threshold of 40% [14].

The trend of change in rumination and job embeddedness at three time points

As for the overall trend (T1-T3), rumination decreased significantly from (50.21±5.14) at baseline to (43.27±5.23) (F=48.050, P<0.001). The score of job embeddedness increased significantly from (20.05±3.10) to (25.36±3.88) (F=25.714, P<0.001), as shown in Table 2, Figure 1 and Figure 2.

Table 2 Dynamic changes of rumination and job embeddedness of trainee nurses during internship (*n*=382)

Variables	T1	T2	T3	F/χ^2	P
**Rumination**					
Total score ($\bar{x} \pm s$)	45.14±5.24	43.36±6.06	41.45±5.22	48.050	< 0.001
Percentage of grading [*n* (%)]				26.903	< 0.001
Low (22 to 33 points)	52 (13.61)	84 (21.99)	101 (26.44)		
Medium (34 to 66)	246 (64.40)	237 (61.04)	234 (61.26)		
High (37 to 88 points)	84 (21.99)	61 (15.97)	47 (12.30)		
**Job embeddings**					
Total score ($\bar{x} \pm s$)	22.07±4.87	23.63±4.85	25.38±4.62	25.714	< 0.001
Percentage of grading [*n* (%)]				34.488	< 0.001
Low (7-14 points)	75 (19.63)	60 (15.71)	49 (12.83)		

Low (15 to 21 points)	135 (35.34)	101 (26.44)	92 (24.08)
Higher (22 to 28 points)	151 (39.53)	174 (45.55)	185 (48.43)
High (29 to 35 points)	21 (5.50)	47 (12.30)	56 (14.66)

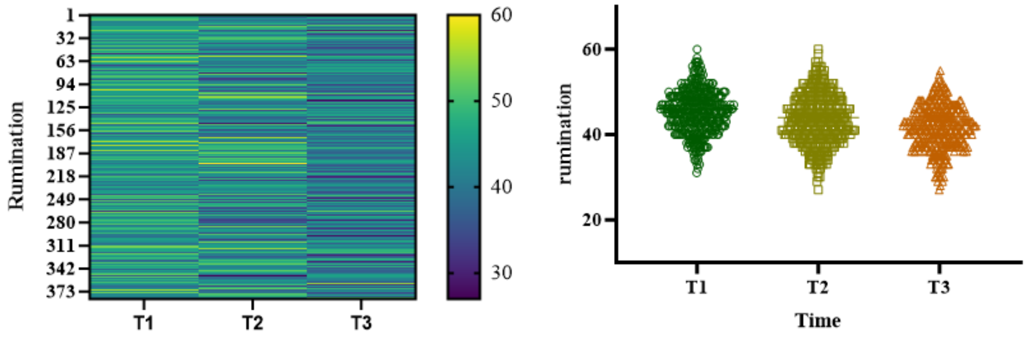

Figure 1 Heat map distribution and trend map of rumination of trainee nurses during internship

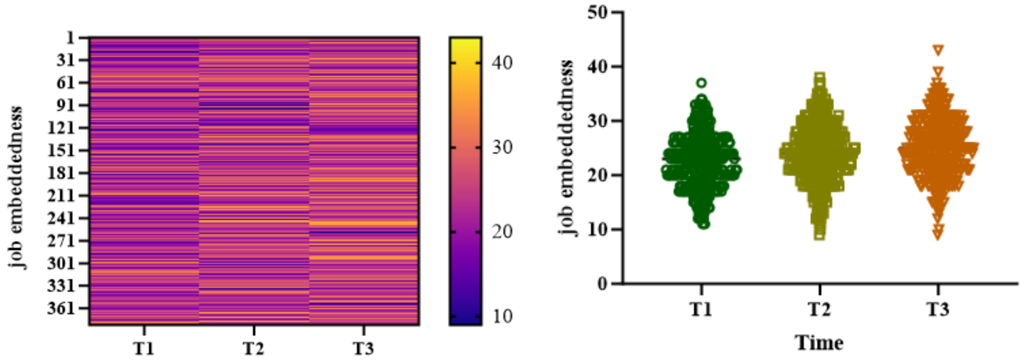

Figure 2 Heat map distribution and trend of job embeddedness heatmap of trainee nurses during internship

Correlation Analysis of Rumination and Job Embeddedness Scores at Three Time Points

Pearson correlation analysis was utilized to examine the relationship between rumination and job embeddedness at three distinct time points. The results indicated a significant correlation between the two variables at all three time points ($P < 0.05$), fulfilling the prerequisites for both the cross-lag model and the latent variable growth model. The matrix representation of these relationships is presented in Table 3.

Table 3 Correlation coefficient matrix of rumination and job embeddedness of trainee nurses at three time points (r, n=382)

Items	①	②	③	④	⑤	⑥
①Rumination T1	1					
②Job embeddedness T1	-0.439**	1				
③Rumination T2	0.542**	-0.337*	1			
④Work embedded in T2	-0.401**	0.482**	-0.472**	1		
⑤Rumination T3	0.462**	-0.305*	0.514**	-0.275*	1	
⑥Work embedding T3	-0.303*	0.370**	-0.347**	0.493**	-0.434***	1

Note:* denotes P<0.05, **denotes P<0.01, and *** P<0.001

Cross-lagged model of rumination and job embeddedness in trainee nurses

Four path models (M1-M4) were constructed to examine the causal relationship between rumination and job embeddedness: (1) M1 (baseline model) featured an autoregressive path (T1→T2→T3) that included only rumination and job embeddedness. M2 (one-way prediction model) built upon M1 by adding the prediction path of rumination on job embeddedness at the subsequent time point (T1_rumination→T2_job embeddedness, T2_rumination→T3_job embeddedness); M3 (reverse prediction model) also expanded upon M1 by incorporating the prediction path of job embeddedness influencing rumination at the next time point (T1_job embeddedness→T2_rumination, T2_job embeddedness→T3_rumination); M4 (two-way full model) encompassed both bidirectional paths of rumination→job embeddedness and job embeddedness→rumination. Robust maximum likelihood estimation (MLR) was utilized, and model fit comparisons are detailed in Table 4. Notably, M4 significantly outperformed M1, M2, and

M3 ($\Delta\chi^2$=137.228, P<0.001), and exhibited the best fit (CFI>0.984, RMSEA<0.05), lending support to the hypothesis of their interaction.

Table 4 Model fitting comparison

Model	χ^2/df	CFI	TLI	RMSEA	SRMR	$\Delta\chi^2$ (vs M1)	Δ df	P
M1	2.350	0.932	0.914	0.078	0.069			
M2	1.814	0.960	0.942	0.064	0.048	125.123	2	<0.001
M3	2.133	0.955	0.934	0.067	0.054	20.560	2	<0.001
M4	1.417	0.984	0.978	0.040	0.034	137.228	4	<0.001

A CLM was constructed to examine the reciprocal predictive relationship between rumination and job embeddedness. The model demonstrated a good fit: χ^2/df = 2.034, CFI = 0.986, TLI = 0.972, RMSEA = 0.000, and SRMR = 0.037. As shown in Figure 1, rumination at the previous time point significantly and negatively predicted job embeddedness at the subsequent time point (β = -0.403, P < 0.01; β = -0.411, P < 0.001). Similarly, job embeddedness at the prior time point significantly and negatively predicted rumination at the next time point (β = -0.397, P < 0.001; β = -0.385, P < 0.001). The detailed path relationships are illustrated in Figure 3.

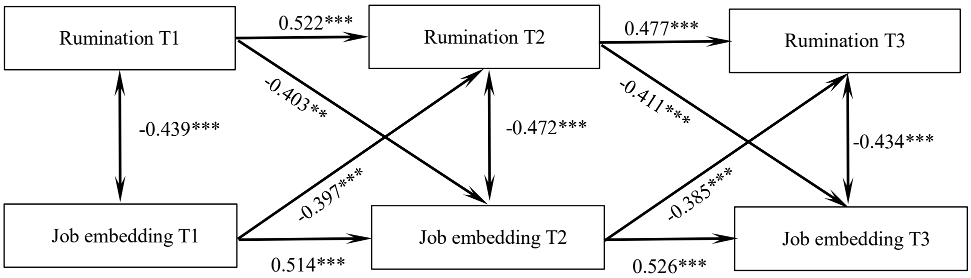

Figure 3 Predictive pathways of rumination and job embedding of trainee nurses at three time points

Note: ** denotes P<0.01 and *** denotes P<0.001

trainee nurses' rumination and job embeddedness in parallel latent variables

A parallel latent variable growth model was constructed to examine the longitudinal relationship between rumination and job embeddedness among trainee nurses. The model demonstrated a good fit: $\chi^2/df=2.062$, CFI=0.984, TLI=0.972, RMSEA=0.048, and SRMR=0.030. At the initial level, rumination significantly and negatively predicted job embeddedness (β=-0.433, *P*=0.002), indicating that trainee nurses with higher initial levels of rumination exhibited lower initial levels of job embeddedness. Additionally, the initial level of rumination negatively predicted the growth rate of job embeddedness (β=-0.395, *P*=0.005), suggesting that higher baseline rumination was associated with a slower increase in job embeddedness over time. Furthermore, the rate of change in rumination positively predicted the rate of change in job embeddedness (β=0.450, *P* < 0.001), meaning that a faster decline in rumination was associated with a more rapid increase in job embeddedness. The specific path relationships are illustrated in Figure 4.

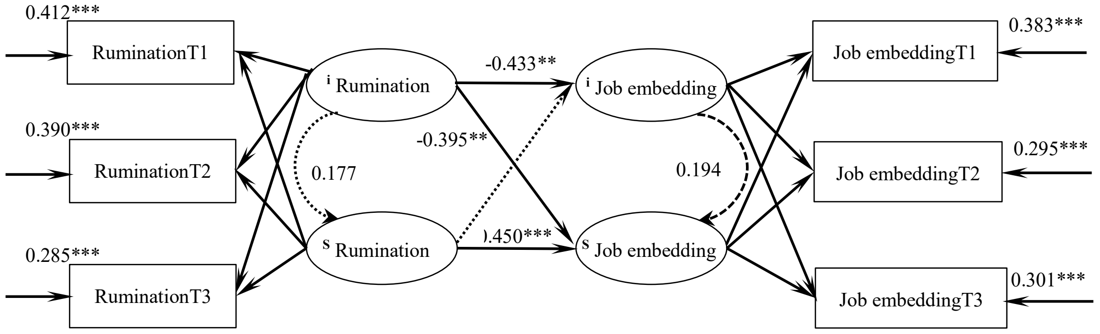

Figure 4 Parallel latent variable model of Rumination and job embeddedness for trainee nurses

Note:** indicates P<0.01 and *** indicates P<0.001

Discussion

This longitudinal study found that among college trainee nurses, rumination decreased while job

embeddedness increased during clinical practice. The clinical practice environment plays a crucial role in shaping these changes. Bhuiya et al.[15] reported that supportive clinical guidance and a positive team atmosphere significantly enhance trainee nurses' self-confidence and sense of belonging, reducing negative emotions during work. The professional qualities and enthusiasm of experienced nurses and clinical instructors subtly influence trainee nurses [16]. Through observation and practice, trainee nurses internalize these behaviors, developing positive professional norms and attitudes [17]. Consequently, rumination declines and job embeddedness rises over the internship period. The internship offers trainee nurses opportunities to engage in nursing work and experience the fulfillment of helping Participants. Moreover, the guidance and role modeling by clinical teachers deepen their understanding of the nursing profession's value [18, 19]. Caring for Participants and addressing nursing challenges further strengthens their professional identity and sense of mission, which clarifies their understanding of nursing and reinforces their commitment to the profession, reflected in increasing job embeddedness scores.

Using a cross-lag model, this study found that higher rumination at an earlier time point negatively predicted job embeddedness at a later time, while higher job embeddedness similarly predicted lower rumination at the subsequent time point. From the perspective of cognitive resource competition, rumination fosters indulgence in negative emotions, hindering professional role cognition and weakening organizational commitment weakening[5]. Conversely, job embeddedness enhances professional identity, directing cognitive resources toward professional engagement and alleviating rumination. Emotionally, a high level of job embeddedness provides positive experiences that interrupt the negative cycle of rumination [20, 21].

The parallel latent variable growth model showed that at the initial level, rumination negatively

predicted job embeddedness; in other words, the higher the initial rumination of trainee nurses, the lower their initial job embeddedness. High rumination accelerates the loss of professional enthusiasm due to ongoing emotional exhaustion, which hinders cognitive reconstruction of professional roles, causing doubts about nursing work and reducing job embeddedness. Additionally, the initial level of rumination negatively predicted the development rate of job embeddedness, higher initial rumination corresponded to a slower increase in job embeddedness. Trainee nurses with elevated rumination tend to engage in negative thinking, such as repeatedly recalling operational errors, which forms a rigid cognitive pattern of rumination and self-denial. This leads to a continuous decline in self-efficacy and reduced willingness to engage in work. Excessive rumination also consumes cognitive resources, making it harder to manage clinical challenges effectively and impeding the development of professional competence. Moreover, rumination-induced negative emotions lower sensitivity to organizational support, reduce proactive seeking of mentor or peer guidance, and delay the formation of professional identity and organizational commitment, thus slowing job embeddedness growth. Conversely, the development speed of rumination positively predicted the development speed of job embeddedness: the faster rumination decreased, the faster job embeddedness increased. As rumination declines, trainee nurses are gradually freed from negative thinking, allowing cognitive resources to be redirected toward career growth and enhancing self-efficacy. Improved emotional regulation also boosts career engagement willingness and encourages active participation in clinical practice, accelerating the process of job embeddedness process [22].

Conclusions

Rumination among trainee nurses showed a downward trend, while job embeddedness showed an

upward trend. The initial level of rumination negatively predicted both the initial level and the development speed of job embeddedness. Additionally, the development speed of rumination positively predicted the development speed of job embeddedness. College and clinical instructors should monitor the rumination levels of trainee nurses and strategically leverage the interaction between rumination and job embeddedness to enhance job embeddedness and strengthen professional identity. This study’s longitudinal design tracked changes in rumination and job embeddedness in 382 trainee nurses throughout their internship, providing valuable insights for clinical understanding of their development. However, some limitations exist: despite being a multi-center study, the relatively small sample size may reduce statistical power.

Conflict of Interest

The study was supported by Project of China Hospital Reform and Development Research Institute of Nanjing University ,Nanjing Drum Tower Hospital and Aid project of Jiangsu Ningai Medical Development & Medical Aid Foundation(NDYGN2024064).

Informed consent was obtained from all Participants in this study.

The authors have no conflicts of interest to disclose.

This study was finally approved by the Ethics Committee of Nanjing Drum Tower Hospital(ID: 2023-0124-02)

Data availability:

Data available on request from the authors.

The data that support the findings of this study are available from the corresponding author, [Chen

Ying], upon reasonable request.

References

[1] Avraham R, Wacht O, Yaffe E, et al. Choosing a Nursing Career During a Global Health Event: A Repeated Cross-Sectional Study[J]. Nurse Educ, 2023,48(4):E116-E121. (in Chinese with English abstract)

[2] Thennakoon S, Ang S, Traynor V, et al. An Integrative Review of Specialised Nursing Career Frameworks to Develop a Nursing Career Framework for Registered Nurses Working in Aged Care[J]. J Adv Nurs, 2024.

[3] Liu M, Wang N, Wang P, et al. Negative Emotions and Job Burnout in News Media Workers: [3] A Moderated Mediation Model of Rumination and Empathy[J]. J Affect Disord, 2021,279:75-82.

[4] Ge W J, Zhu S J, Zhu X Y, et al. Gender differences in co-rumination and transition shock among nursing interns in China: a cross-sectional study[J]. BMC Nurs, 2020,24(1):420.

[5] Li C, Wu M, Zeng L, et al. The chain mediating role of rumination and social withdrawal in the relationship between smartphone distraction and depression among Chinese trainee nurses[J]. Sci Rep, 2024,14(1):18116.

[6] Atta M, El-Ashry A M, Abd E M M. The effects of mindfulness-based techniques on self-rumination, cognitive reappraisal and expressive suppression among Participants with major depression: a nursing perspective[J]. J Res Nurs, 2024,29(4-5):366-385.

[7] Zeng L, Wang J, Liu G, et al. Rumination, psychological capital and academic procrastination among nursing students: A cross-sectional study[J]. Nurse Educ Today, 2024,137:106170.

[8] Thomson E. Commentary: The effects of mindfulness-based techniques on self-rumination,

cognitive reappraisal and expressive suppression among Participants with major depression: a nursing perspective[J]. J Res Nurs, 2024,29(4-5):386-387.

[9] El-Sayed M M, Hawash M M, Khedr M A, et al. Golden steps: A nursing approach for vitality, agility, and rumination in geriatric clients with depressive disorders through mindful walking[J]. Arch Psychiatr Nurs, 2024 ploidy: 212-221.

[10] Geisler M, Buratti S, Allwood C M. Affective work rumination as a mediator of the reciprocal relationships between job demands and exhaustion[J]. PLoS One, 2023,18(11):e293837.

[11] Han Xiu, Yang Hong-fei. Application of Nolen-Hoeksema Ruminant Thinking Scale in China [J]. Chinese Journal of Clinical Psychology, 2009(5).

[12] Crossley C D, Bennett R J, Jex S M, et al. Development of a global measure of job embeddedding and integration into a traditional model of voluntary turnover[J]. J Appl Psychol, 2007,92(4):1031-1042.

[13] Mei Hua, Qin Xiufang, Cui Yi, et al. [13] Mei Mei, QIN Xiufang, Cui Yi, et al. Correlation between job embeddedding and turnover intention of nurses in Shanghai Traditional Chinese Medicine hospital [J]. Journal of Nursing Management, 2014,14(5):305-307.

[14] Tang Dandan, Wen Zhong-lin. [14] TANG Dantan, WENG Zhonglin. Common Method bias testing: Problems and suggestions [J]. Psychological Science, 2020,43(1):215-223.

[15] Bhuiya T, Zhong X, Pollack G, et al. Tell Me More(R): A medical student focused humanistic communication model to enhance student professional identity formation through meaningful patient encounters[J]. Patient Educ Couns, 2021,105(3):641-646.

[16] Burcul V, Chartrand J, Balasa R, et al. An exploration of trainee nurses' and clinical instructors' perceptions of patient and parent involvement in the assessment of trainee nurses during

pediatric clinical placements: A qualitative study[J]. Nurse Educ Pract, 2021,56:103195.

[17] Villa G, Galli E, Allieri S, et al. Frontline Involvement in Population COVID-19 Vaccinations: Lived Experience of trainee nurses[J]. Healthcare (Basel), 2021,10(10).

[18] Loura D S, Bernardes R A, Baixinho C L, et al. trainee nurses' learning from involvement in research projects: an integrative literature review[J]. Rev Bras Enferm, 2021,75(1):e20210053.

[19] Suikkala A, Koskinen S, Leino-Kilpi H. Participants' involvement in trainee nurses' clinical education: A scoping review[J]. Int J Nurs Stud, 2018,84:40-51.

[20] Mitropoulos T, Andrukonis A. Dog owners' job stress crosses over to their pet dogs via work-related rumination[J]. Sci Rep, 2025, 15 (1) : 16887.

[21] Liu J, Wang W, Hu Q, et al. The relationship between phubbing and the depression of primary and secondary school teachers: A moderated mediation model of rumination and job burnout[J]. J Affect Disord, 2021,295:498-504.

[22] Weigelt O, Syrek C J, Schmitt A, et al. Finding peace of mind when there still is so much left undone-A diary study on how job stress, competence need satisfaction, and proactive work behavior contribute to work-related rumination during the weekend[J]. J Occup Health Psychol, 2019, 24 (3) : 373-386.

Click here to access/download
Supporting Information
renamed_91885.xlsx
