# Supplementary material for: The interaction between rumination and job embeddedness among trainee nurses during internship: A multicenter longitudinal study
Source: PLoS One. 2026 May 26;21(5):e0349402. doi: 10.1371/journal.pone.0349402 (PMC13210194; doi:10.1371/journal.pone.0349402)
Supplement: Attachment — Submitted filename: Response to Reviewers.docx [file pone.0349402.s003.docx]

Dear Editors and Reviewers,

On behalf of our research team, I would like to express our sincere gratitude for your valuable time and insightful comments on our manuscript. We have carefully addressed each point raised in the review and have revised the manuscript accordingly. We sincerely hope that our responses and revisions meet with your approval.

Yours sincerely,

Weiyi Wang

View Letter

Date:	Jan 05 2026 01:08AM

To:	"weiyi wang" anotherweiyi@163.com

From:	"PLOS ONE" plosone@plos.org

Subject:	PLOS ONE Decision: Revision required [PONE-D-25-60904]

Attachment(s):	 PONE-D-25-60904 (R).pdf

PONE-D-25-60904

The interaction between rumination and job embeddedness among trainee nurses duringinternship: a multicenter longitudinal study

PLOS One

Dear Dr. wang,

Thank you for submitting your manuscript to PLOS ONE. After careful consideration, we feel that it has merit but does not fully meet PLOS ONE’s publication criteria as it currently stands. Therefore, we invite you to submit a revised version of the manuscript that addresses the points raised during the review process.

Please submit your revised manuscript by Feb 19 2026 11:59PM. If you will need more time than this to complete your revisions, please reply to this message or contact the journal office at plosone@plos.org. When you're ready to submit your revision, log on to https://www.editorialmanager.com/pone/ and select the 'Submissions Needing Revision' folder to locate your manuscript file.

Please include the following items when submitting your revised manuscript:

A letter that responds to each point raised by the academic editor and reviewer(s). You should upload this letter as a separate file labeled 'Response to Reviewers'.

A marked-up copy of your manuscript that highlights changes made to the original version. You should upload this as a separate file labeled 'Revised Manuscript with Track Changes'.

An unmarked version of your revised paper without tracked changes. You should upload this as a separate file labeled 'Manuscript'.

If applicable, we recommend that you deposit your laboratory protocols in protocols.io to enhance the reproducibility of your results. Protocols.io assigns your protocol its own identifier (DOI) so that it can be cited independently in the future. For instructions see: https://journals.plos.org/plosone/s/submission-guidelines#loc-laboratory-protocols. Additionally, PLOS ONE offers an option for publishing peer-reviewed Lab Protocol articles, which describe protocols hosted on protocols.io. Read more information on sharing protocols at https://plos.org/protocols?utm_medium=editorial-email&utm_source=authorletters&utm_campaign=protocols.

We look forward to receiving your revised manuscript.

Kind regards,

Ahmed Abdelwahab Ibrahim El-Sayed

Academic Editor

PLOS One

Journal Requirements:

When submitting your revision, we need you to address these additional requirements.

1. Please ensure that your manuscript meets PLOS ONE's style requirements, including those for file naming. The PLOS ONE style templates can be found at

https://journals.plos.org/plosone/s/file?id=wjVg/PLOSOne_formatting_sample_main_body.pdf and https://journals.plos.org/plosone/s/file?id=ba62/PLOSOne_formatting_sample_title_authors_affiliations.pdf

2. Thank you for stating the following financial disclosure:

“The study was supported by Project of China Hospital Reform and Development Research Institute of Nanjing University ,Nanjing Drum Tower Hospital and Aid project of Jiangsu Ningai Medical Development & Medical Aid Foundation(NDYGN2024064).”

Please state what role the funders took in the study. If the funders had no role, please state: "The funders had no role in study design, data collection and analysis, decision to publish, or preparation of the manuscript."

If this statement is not correct you must amend it as needed.

Please include this amended Role of Funder statement in your cover letter; we will change the online submission form on your behalf.

Response: This point has been added to the manuscript.

3. Thank you for providing your underlying data as Supporting Information.

We note that the data set contains text or data that is not in English. Please note that PLOS is an English-language publisher, so we require data sets to be provided in English as well. Please upload an English-language version of your data set.

This will also allow us to determine if your data follows PLOS standards per our Data Availability policy here: <https://journals.plos.org/plosone/s/data-availability>

Response: Data has been updated.

4. Your ethics statement should only appear in the Methods section of your manuscript. If your ethics statement is written in any section besides the Methods, please move it to the Methods section and delete it from any other section. Please ensure that your ethics statement is included in your manuscript, as the ethics statement entered into the online submission form will not be published alongside your manuscript.

Response: This point has been addressed in the manuscript.

5. Please include captions for your Supporting Information files at the end of your manuscript, and update any in-text citations to match accordingly. Please see our Supporting Information guidelines for more information: http://journals.plos.org/plosone/s/supporting-information.

6. If the reviewer comments include a recommendation to cite specific previously published works, please review and evaluate these publications to determine whether they are relevant and should be cited. There is no requirement to cite these works unless the editor has indicated otherwise.

Additional Editor Comments:

Dear Authors,

Thank you for submitting your manuscript to PLOS ONE. Based on the reviewers’ evaluations, several substantive concerns have been raised that must be thoroughly addressed before further consideration of your manuscript.

We encourage you to carefully review the comments and submit a revised version that responds to each point.

[Note: HTML markup is below. Please do not edit.]

Reviewers' comments:

Reviewer's Responses to Questions

Comments to the Author

1. Is the manuscript technically sound, and do the data support the conclusions?

The manuscript must describe a technically sound piece of scientific research with data that supports the conclusions. Experiments must have been conducted rigorously, with appropriate controls, replication, and sample sizes. The conclusions must be drawn appropriately based on the data presented.

Reviewer #1: Partly

Reviewer #2: Yes

Reviewer #3: Yes

Reviewer #4: Yes

2. Has the statistical analysis been performed appropriately and rigorously?

Reviewer #1: Yes

Reviewer #2: Yes

Reviewer #3: Yes

Reviewer #4: Yes

3. Have the authors made all data underlying the findings in their manuscript fully available?

The PLOS Data policy requires authors to make all data underlying the findings described in their manuscript fully available without restriction, with rare exception (please refer to the Data Availability Statement in the manuscript PDF file). The data should be provided as part of the manuscript or its supporting information, or deposited to a public repository. For example, in addition to summary statistics, the data points behind means, medians and variance measures should be available. If there are restrictions on publicly sharing data—e.g. participant privacy or use of data from a third party—those must be specified.

Reviewer #1: Yes

Reviewer #2: Yes

Reviewer #3: Yes

Reviewer #4: No

4. Is the manuscript presented in an intelligible fashion and written in standard English?

PLOS ONE does not copyedit accepted manuscripts, so the language in submitted articles must be clear, correct, and unambiguous. Any typographical or grammatical errors should be corrected at revision, so please note any specific errors here.

Reviewer #1: Yes

Reviewer #2: Yes

Reviewer #3: Yes

Reviewer #4: No

5. Review Comments to the Author

Please use the space provided to explain your answers to the questions above. You may also include additional comments for the author, including concerns about dual publication, research ethics, or publication ethics. (Please upload your review as an attachment if it exceeds 20,000 characters)

Reviewer #1: Title: The interaction between rumination and job embeddedness among trainee nurses during internship: a multicenter longitudinal study.

I. Overall

II. Abstract

1. Although this is underpinned by the three key moments you described later, I think that clearly mentioning the study design is a non-negotiable requirement.

Response: The supplementary content has been added to the abstract section.

2. Please specify the instruments used in the abstract.

Response: The necessary revisions have been made to the abstract.

3. Keywords: Please, use the exact MeSH terms for the "Keywords" section to ensure better indexing.

Response: The revisions have been made as requested.

III. Introduction

1. Line 44 ► What do you mean by “participants”?

Response: The term has been changed to 'patients'.

2. Lines 48-50 ►“During clinical practice […] first time [4]”: I think that reference [4] does not support this claim.

3. Lines 50-53 ►“The transition […] practice stage [5]”: I think that reference [5] does not support this claim.

4. Lines 54-56 ►“Research has shown […] attentional resources [6]”: I think that reference [6] does not claim.

5. Lines 58-62 ►“More critically […] nursing talent echelon [8, 9]”: I think that reference [8,9] does NOT support the claim you provided, because both references refer to patients, not nursing students.

6. Lines 63-65 ►“Understanding the developmental trajectory […] job embeddedness [10]”: Since the reference [10] never measured job embeddedness, it cannot statistically support a claim about enhancing it.

7. Please revise the introduction to ensure every factual claim and contextual statement is supported by a robust, peer-reviewed source. This is critical for academic rigor and for accurately framing the research gap your work aims to address.

Response: Thank you for your feedback. We have verified and revised the relevant references accordingly.

8. Lines 72-74 ► Lines 63-65 ► I think that your study is an empirical study, the phrase "aims to provide a theoretical foundation" might be slightly overstating the primary goal.

Response: The relevant wording has been revised.

IV. Materials and methods

1. In adherence to the journal’s submission guidelines, please revise the section heading ‘Research methods’ to ‘Materials and Methods’.

Response: The revisions have been made as requested.

2. Reorganize your Methods section with clear, non-overlapping subheadings.

Response: The revisions have been made as requested.

3. The claim that "at least 200 cases were needed" for the LGM must be supported.

Response: The references have been added.

4. For an LGM, a simulation-based power analysis is the gold standard. Please provide details: target parameter, assumed effect size (with justification from prior literature), number of waves, and software/method used. Without this, the sample size is arbitrary.

Response: The revisions have been made as requested.

5. Please provide a citation or empirical rationale for the anticipated 20% attrition rate.

Response: The revisions have been made as requested.

V. Results

1. Table 2 shows an inconsistency in the percentage of rating categories.

Response: The data has been verified.

2. What is your reference for establishing the rating categories for the two scales? Normally, these categories should be defined in the methods.

Response: The evaluation criteria have been added at the introduction section of the scale.

3. Reference [13] is included in the list of references, but is not cited in the text.

Response: The full list of references has been verified.

4. Figure 4 requires a legend to facilitate its reading.

Response: It has been supplemented in the article.

VI. Discussion

1. Lines 207-209 ► “Bhuiya et al. [15] […] during work”: This claim does not belong to the reference [15]. The latter is about medical students, not nurses.

2. Lines 212-215 ►“Consequently […] nursing profession’s value [18,19 ]: It appears that reference [18] is entirely fictitious.

3. Lines 216-218 ►“Caring for Participants […] job embeddedness scores”: This claim need to be supported by at least one reference.

4. Lines 221-223 ►“From the perspective […] commitment weakening [5]”: This claim is not supported by the reference [5]. Actually, the key finding of the latter is that rumination is not only a direct pathway to depression but also a trigger for social withdrawal, which further deepens depressive symptoms.

5. Lines 225-226 ►“Emotionally […] negative cycle of rumination [20, 21]”: The two cited references do not provide support for the concept of "job embeddedness," as they do not investigate this construct. To support this claim, please cite literature that specifically defines and examines job embeddedness.

Response: References have been verified and updated.

6. Lines 234-247 ►“Trainee nurses […] job embeddedness process [22]”: This 15-line discussion passage contains overinterpretation, distortion of facts, or speculation presented as evidence.

Response: The relevant expressions have been revised.

7. Overall, the discussion section lacks synthesis and scholarly depth. I strongly advise you to review the discussion section in question and ensure every sentence that engages with a source can be directly and justifiably traced back to that source’s actual content.

Response: Thank you for your review. The discussion content has been revised.

VII. References

1. “Meihua et al.” is cited in the text but absent from the reference list.

2. “Crossley et al. ” is formatted incorrectly in the text per the journal style.

3. What does the symbol [J] at the end of the titles mean?

4. It would be wise to include the DOI when possible.

5. Please verify the title of the reference [3].

6. The entries for these references are incomplete (eg. [11], [14], [17]).

7. It appears that reference [18] is entirely fictitious.

Response: Thank you for your meticulous review. I have made the necessary revisions as requested.

Reviewer #2: The revised manuscript entitled “The interaction between rumination and job embeddedness among trainee nurses during internship: a multicenter longitudinal study” is generally well structured and clearly written. The introduction is informative; however, the authors should carefully verify that the references cited are fully consistent with the statements made. The methodology is clearly described, although the sampling method should be explicitly stated. The results are well presented and appropriately discussed. In addition, the limitations section should be expanded to include aspects beyond the small sample size. Finally, the authors are encouraged to carefully check and standardize all references according to the Vancouver referencing style.

Detailed comments and specific remarks are provided in the attached file.

Response: Thank you for your meticulous review. I have made the necessary revisions as requested.

Reviewer #3: O manuscrito é tecnicamente sólido por apresentar um delineamento longitudinal adequado, com a coleta de dados realizada em três momentos, com uso de abordagens estatísticas robustas e confiáveis (Modelo de defazagem cruzada e modelo paralelo de crescimento latente). A descrição dos métodos foi realizada de maneira clara e apropriada para as respostas as questões da pesquisa. As análises estatísticas forão rigorosas e apresentadas de forma bem transparente. Os indices de ajuste dos modelos utilizados forão reportados adequadamente, e o teste de viés reforçou a confiabilidade dos resultados.

É uma pesquisa original e de relevancia por analisar a ruminação e o enraizamento no trabalho em enfermeiros estagiários, devido principalmente ao contexto de formação clínica.

As conclusões vão de encontro aos dados apresentados , todas as afirmações são sustentadas. O manuscrito é claro, bem estruturado e intelegível para todos, as tabelas são pertinentes e contribuem para compreensão dos achados.

Foi apresentada a aprovação do Comitê de Ética e Pesquisa, e o estudo informou a disponibilidade dos dados de acordo com as diretrizes da revista.

Algumas considerações: Sugiro embasar mais a discussão articulando com modelos teóricos clássicos e contempoâneos sobre o estresse ocupacional, adaptação profissional e saúde mental dos profissionais de saúde. Falar um pouco mais sobre as características culturais e organizacionais do contexto chinês, acrescentar exemplos específicos de intervenções institucionais aplicaveis ao programa de estágio em enfermagem.

Realizar pequenos ajustes na linguagem para melhorar a fluidez, em trechos maiores. Garantir que todas as siglas e abreviações estatísticas sejam definidas na primeira menção no texto.

Response: Agradeço pela sua revisão meticulosa. Realizei as revisões necessárias conforme solicitado.

Reviewer #4: The study presents a sound longitudinal design and an appropriate use of advanced statistical models; however, its contribution to disciplinary knowledge in nursing is limited. The inverse relationship between rumination and job embeddedness has already been widely documented in the literature, particularly in contexts where employability expectations influence the psychological wellbeing of nurses in training. The findings largely confirm predictable trends without offering new conceptual frameworks, additional explanatory variables, or innovative practical implications for nursing education or management. Furthermore, the construct of rumination is not sufficiently contextualised from a nursing perspective. Overall, the study stands out more for its methodological rigour than for making a substantive contribution to the advancement of nursing knowledge.

Response: Thank you for your meticulous review. I have made the necessary revisions as requested.

6. PLOS authors have the option to publish the peer review history of their article (what does this mean?). If published, this will include your full peer review and any attached files.

If you choose “no”, your identity will remain anonymous but your review may still be made public.

Do you want your identity to be public for this peer review? For information about this choice, including consent withdrawal, please see our Privacy Policy.

Reviewer #1: No

Reviewer #2: Yes: EL FADELY ABDELMONIM

Reviewer #3: No

Reviewer #4: No

[NOTE: If reviewer comments were submitted as an attachment file, they will be attached to this email and accessible via the submission site. Please log into your account, locate the manuscript record, and check for the action link "View Attachments". If this link does not appear, there are no attachment files.]

To ensure your figures meet our technical requirements, please review our figure guidelines: https://journals.plos.org/plosone/s/figures

You may also use PLOS’s free figure tool, NAAS, to help you prepare publication quality figures: https://journals.plos.org/plosone/s/figures#loc-tools-for-figure-preparation.

NAAS will assess whether your figures meet our technical requirements by comparing each figure against our figure specifications.

In compliance with data protection regulations, you may request that we remove your personal registration details at any time. (Remove my information/details). Please contact the publication office if you have any questions.
