# Supplementary material for: The interaction between rumination and job embeddedness among trainee nurses during internship: A multicenter longitudinal study
Source: PLoS One. 2026 May 26;21(5):e0349402. doi: 10.1371/journal.pone.0349402 (PMC13210194; doi:10.1371/journal.pone.0349402)
Supplement: Attachment — Submitted filename: Response_to_Reviewers_auresp_2.docx [file pone.0349402.s004.docx]

View Letter

Date:	Feb 26 2026 08:27PM

To:	"Chen Ying" 13814021690@163.com

From:	"PLOS ONE" plosone@plos.org

Subject:	PLOS ONE Decision: Revision required [PONE-D-25-60904R1]

PONE-D-25-60904R1

The interaction between rumination and job embeddedness among trainee nurses duringinternship: a multicenter longitudinal study

PLOS One

Dear Dr. Ying,

Thank you for submitting your manuscript to PLOS ONE. After careful consideration, we feel that it has merit but does not fully meet PLOS ONE’s publication criteria as it currently stands. Therefore, we invite you to submit a revised version of the manuscript that addresses the points raised during the review process.

Please submit your revised manuscript by Apr 12 2026 11:59PM. If you will need more time than this to complete your revisions, please reply to this message or contact the journal office at plosone@plos.org. When you're ready to submit your revision, log on to https://www.editorialmanager.com/pone/ and select the 'Submissions Needing Revision' folder to locate your manuscript file.

Please include the following items when submitting your revised manuscript:

A letter that responds to each point raised by the academic editor and reviewer(s). You should upload this letter as a separate file labeled 'Response to Reviewers'.

A marked-up copy of your manuscript that highlights changes made to the original version. You should upload this as a separate file labeled 'Revised Manuscript with Track Changes'.

An unmarked version of your revised paper without tracked changes. You should upload this as a separate file labeled 'Manuscript'.

If you would like to make changes to your financial disclosure, please include your updated statement in your cover letter. Guidelines for resubmitting your figure files are available below the reviewer comments at the end of this letter.

If applicable, we recommend that you deposit your laboratory protocols in protocols.io to enhance the reproducibility of your results. Protocols.io assigns your protocol its own identifier (DOI) so that it can be cited independently in the future. For instructions see: https://journals.plos.org/plosone/s/submission-guidelines#loc-laboratory-protocols. Additionally, PLOS ONE offers an option for publishing peer-reviewed Lab Protocol articles, which describe protocols hosted on protocols.io. Read more information on sharing protocols at https://plos.org/protocols?utm_medium=editorial-email&utm_source=authorletters&utm_campaign=protocols.

We look forward to receiving your revised manuscript.

Kind regards,

Ahmed Abdelwahab Ibrahim El-Sayed

Academic Editor

PLOS One

Journal Requirements:

1. If the reviewer comments include a recommendation to cite specific previously published works, please review and evaluate these publications to determine whether they are relevant and should be cited. There is no requirement to cite these works unless the editor has indicated otherwise.

2. Please review your reference list to ensure that it is complete and correct. If you have cited papers that have been retracted, please include the rationale for doing so in the manuscript text, or remove these references and replace them with relevant current references. Any changes to the reference list should be mentioned in the rebuttal letter that accompanies your revised manuscript. If you need to cite a retracted article, indicate the article’s retracted status in the References list and also include a citation and full reference for the retraction notice.

Additional Editor Comments:

Dear Authors,

Thank you for your revision. We appreciate the improvements made to the manuscript. However, in this round of review, the reviewer has raised some minor comments that need to be addressed carefully before we can proceed to a final decision regarding your study.

Please revise the manuscript accordingly and provide a detailed response to the reviewer’s comments.

[Note: HTML markup is below. Please do not edit.]

Reviewers' comments:

Reviewer's Responses to Questions

Comments to the Author

1. If the authors have adequately addressed your comments raised in a previous round of review and you feel that this manuscript is now acceptable for publication, you may indicate that here to bypass the “Comments to the Author” section, enter your conflict of interest statement in the “Confidential to Editor” section, and submit your "Accept" recommendation.

Reviewer #1: All comments have been addressed

Reviewer #2: (No Response)

Reviewer #3: All comments have been addressed

Reviewer #4: All comments have been addressed

2. Is the manuscript technically sound, and do the data support the conclusions?

The manuscript must describe a technically sound piece of scientific research with data that supports the conclusions. Experiments must have been conducted rigorously, with appropriate controls, replication, and sample sizes. The conclusions must be drawn appropriately based on the data presented.

Reviewer #1: Yes

Reviewer #2: Partly

Reviewer #3: Yes

Reviewer #4: Yes

3. Has the statistical analysis been performed appropriately and rigorously?

Reviewer #1: Yes

Reviewer #2: I Don't Know

Reviewer #3: Yes

Reviewer #4: Yes

4. Have the authors made all data underlying the findings in their manuscript fully available?

The PLOS Data policy requires authors to make all data underlying the findings described in their manuscript fully available without restriction, with rare exception (please refer to the Data Availability Statement in the manuscript PDF file). The data should be provided as part of the manuscript or its supporting information, or deposited to a public repository. For example, in addition to summary statistics, the data points behind means, medians and variance measures should be available. If there are restrictions on publicly sharing data—e.g. participant privacy or use of data from a third party—those must be specified.

Reviewer #1: Yes

Reviewer #2: Yes

Reviewer #3: Yes

Reviewer #4: No

5. Is the manuscript presented in an intelligible fashion and written in standard English?

PLOS ONE does not copyedit accepted manuscripts, so the language in submitted articles must be clear, correct, and unambiguous. Any typographical or grammatical errors should be corrected at revision, so please note any specific errors here.

Reviewer #1: Yes

Reviewer #2: Yes

Reviewer #3: Yes

Reviewer #4: Yes

6. Review Comments to the Author

Please use the space provided to explain your answers to the questions above. You may also include additional comments for the author, including concerns about dual publication, research ethics, or publication ethics. (Please upload your review as an attachment if it exceeds 20,000 characters)

Reviewer #1: (No Response)

Reviewer #2: The following comments are provided to clarify remaining issues that were not fully addressed in the revised manuscript and that require further attention to ensure scientific accuracy and transparency.

1. The reviewer’s previous comment has not been addressed.

The abstract still reports a recovery rate of 93.53% for 405 questionnaires, whereas the Results section clearly indicates that 382 valid questionnaires were collected, corresponding to an effective response rate of 94.32%.

Please correct the percentage reported in the abstract or provide a clear explanation for the discrepancy.

Response: We appreciate the reviewer's thorough comments. Corresponding revisions have been made in the manuscript.

2. The revised manuscript does not adequately address the previous comment.

The statement claiming that “the correlation between persistent rumination and anxiety or depression is 2.5 times higher in trainee nurses than in the general population” remains unsupported by the cited literature.

The newly introduced reference does not report (i) data on trainee nurses, (ii) comparisons with the general population, nor (iii) effect size ratios indicating a 2.3–2.5-fold difference.

As currently written, this claim constitutes an unsubstantiated quantitative assertion and raises concerns regarding scientific accuracy.

Please either provide a source that explicitly reports this population-specific effect size or revise the statement to accurately reflect the available evidence.

Response: We appreciate the reviewer's thorough comments. The corresponding expressions and references have been modified in the manuscript.

3. The reviewer’s previous concern has not been addressed.

The revised manuscript still attributes specific percentage differences (19 % decrease in clinical performance accuracy and 41 % lower professional identity score) to Zeng et al. (2024).

However, this study is cross-sectional, does not include a control group, and does not report outcomes related to clinical performance accuracy or professional identity, nor any percentage-based group comparisons.

Merely changing the reference number does not resolve the issue. Please cite the correct source that explicitly reports these figures or remove/revise the statement accordingly.

Response: We appreciate the reviewer's thorough comments. The relevant expressions have been modified in the manuscript.

4. The authors’ revision does not adequately address the reviewer’s concern.

4.1 Unsupported causal and intervention-related claims

Although the cited reference has been removed, the statement itself remains unchanged. As currently written, the paragraph presents causal and intervention-related claims (e.g., mitigation of negative emotions, enhancement of job embeddedness through psychological interventions) without empirical support.

Removing the citation without revising or qualifying the claim does not resolve the issue. The authors should either provide appropriate evidence supporting these assertions or clearly reformulate the text as a hypothesis or conceptual perspective rather than an evidence-based conclusion.

Response: We appreciate the reviewer's thorough comments. The relevant expressions have been modified in the manuscript.

4.2 Continued inappropriate use of the same reference elsewhere in the manuscript

In addition, it should be noted that the previously removed reference (Geisler et al., 2023) is reused elsewhere in the manuscript in a manner that remains inappropriate. While Geisler et al. (2023) examine affective work rumination in relation to job demands and exhaustion, the study does not assess cognitive resource competition, professional role cognition, or organizational commitment.

These constructs are neither measured nor empirically discussed in the cited article. Therefore, interpretations attributed to this reference should be revised to clearly distinguish theoretical reasoning from empirically supported findings, or an appropriate reference should be provided.

Response: We appreciate the reviewer's suggestion. The text has been modified to reflect a hypothetical tone.

5. The reviewer’s previous comment has not been addressed.

Although the sample size calculation and inclusion/exclusion criteria are clearly described, the manuscript still does not specify the sampling method or recruitment strategy.

It remains unclear whether participants were recruited via convenience sampling, cluster sampling by institution, or another approach, and how the 12 participating institutions and trainee nurses were selected.

Please explicitly state the sampling strategy and recruitment procedure to allow readers to assess potential selection bias and external validity.

Response: We appreciate the reviewer's thorough comments. The relevant expressions have been modified in the manuscript.

6. The revision only partially addresses the reviewer’s comment.

While the statement regarding informed consent has been appropriately removed and the ethics approval ID has been correctly relocated as requested, the “Conflict of Interest” section still includes funding information.

Please separate the funding statement under a dedicated “Funding” subheading and retain only the conflict of interest declaration under “Conflict of Interest,” in line with standard journal structure. Further revision is required to fully address this point.

Response: We appreciate the reviewer's thorough comments. The relevant expressions have been modified in the manuscript.

Reviewer #3: (No Response)

Reviewer #4: This manuscript does not contribute anything new to nursing science from a professional, academic, clinical, or other perspective. However, it is well-written, well-structured, easy to understand, and methodologically reproducible, except for one important flaw: the authors do not provide or make available the study's main component. This component is the self-developed electronic questionnaire distributed through the WenJuanxing platform (survey ID: 23045560). Furthermore, they do not provide the two main scales (RRS and GJEI) that the authors claim are included in the questionnaire. They also fail to state whether this questionnaire has been previously validated, which would be important and would strengthen the study. Therefore, my review focuses on the contribution of the questionnaires to ensure reproducibility, as this would be the study's greatest contribution. The results obtained have already been widely published in many fields and areas.

Response: We appreciate the reviewer's suggestion. The relevant scales, along with validation data on their reliability and validity in China, have been added to the Methods section.

7. PLOS authors have the option to publish the peer review history of their article (what does this mean?). If published, this will include your full peer review and any attached files.

If you choose “no”, your identity will remain anonymous but your review may still be made public.

Do you want your identity to be public for this peer review? For information about this choice, including consent withdrawal, please see our Privacy Policy.

Reviewer #1: No

Reviewer #2: No

Reviewer #3: No

Reviewer #4: Yes: Ana Fernández-Araque

[NOTE: If reviewer comments were submitted as an attachment file, they will be attached to this email and accessible via the submission site. Please log into your account, locate the manuscript record, and check for the action link "View Attachments". If this link does not appear, there are no attachment files.]

To ensure your figures meet our technical requirements, please review our figure guidelines: https://journals.plos.org/plosone/s/figures

You may also use PLOS’s free figure tool, NAAS, to help you prepare publication quality figures: https://journals.plos.org/plosone/s/figures#loc-tools-for-figure-preparation.

NAAS will assess whether your figures meet our technical requirements by comparing each figure against our figure specifications.

In compliance with data protection regulations, you may request that we remove your personal registration details at any time. (Remove my information/details). Please contact the publication office if you have any questions.
